# Multidimensional Urban Exposure Analysis of Industrial Chemical Risk Scenarios in Mexican Metropolitan Areas

**DOI:** 10.3390/ijerph18115674

**Published:** 2021-05-26

**Authors:** Claudia Yazmin Ortega Montoya, Andrés Osvaldo López-Pérez, Marisol Ugalde Monzalvo, Ma. Loecelia Guadalupe Ruvalcaba Sánchez

**Affiliations:** 1 Tecnologico de Monterrey, Escuela de Humanidades y Educación, Torreón 27250, Mexico; yazmin.ortega@tec.mx; 2 Centro de Investigación en Ciencias de Información Geoespacial, Aguascalientes 20313, Mexico; lruvalcaba@centrogeo.edu.mx; 3 Tecnologico de Monterrey, Escuela de Arquitectura y Diseño, Atizapan 52926, Mexico; marisol.ugalde@itesm.mx

**Keywords:** land use planning, risk scenarios, environmental risk analysis, multidimensional spatial analysis

## Abstract

Risk scenarios are caused by the convergence of a hazard with a potentially affected system in a specific place and time. One urban planning goal is to prevent environmental hazards, such as those generated by chemical accidents, from reaching human settlements, as they can cause public health issues. However, in many developing countries, due to their strategic positioning in global value chains, the quick and easy access to labor pools, and competitive production costs, urban sprawls have engulfed industrial areas, exposing residential conurbations to environmental hazards. This case study analyzes the spatial configuration of accidental chemical risk scenarios in three major Mexican metropolitan areas: Mexico City, Guadalajara, and Monterrey. Spatial analyses use an areal locations of hazardous atmosphere (ALOHA) dispersion model to represent the spatial effects of high-risk industrial activities in conurbations and the potentially affected populations vulnerable to chemical hazards. Complementary geostatistical correlation analyses use population data, marginalization indexes, and industrial clustering sectors to identify trends that can lead to comprehensive environmental justice approaches. In addition, the marginalization degree of inhabitants evaluates social inequalities concerning chemical risk scenarios.

## 1. Introduction

The closeness to the market and the availability of goods and energy commonly attract industries to urban areas. A wide variety of industrial processes use chemical substances. This way, chemical flows are vital supplies for economic processes in urban environments [1]. The location of industries and urban areas within city limits depends on diverse scales and factors. To understand the risks involved, we should analyze how societies generate spaces within a specific historical context and what their association is with each production model [2].

Urban planning seeks to favorably influence the spatial and functional organization of cities and regions. Multiple traditions ranging from design to health, law, social action, and economic development have given rise to urban planning. It helps establish the legislative groundwork, agencies, governments, and professionals who legitimize the field and provide a framework for action. In contrast, disaster risk reduction is relatively new [3] and is just recently being incorporated as an urgent factor to consider in urban planning; however, it is not considered in the urban planning of existing cities.

The coexistence between industrial and urban spaces interconnects risk prevention and urban dynamics [4]. Accumulation of hazardous sites is increasing with little regard for shifting neighborhood demographics or existing regulatory policies. As sites merge into larger, more contiguous industrialized areas that generate historical hazards, environmental conditions that put an urban society at risk are created [5]. The government should be aware of these risks so that it can integrate prevention into all planning levels. Risk governance pertains to the many ways in which individuals and institutions (public and private) deal with risks, and it can be transferred to urban planning in the pre-estimation, interdisciplinary estimation, evaluation, and risk management phases [6].

In many countries, the severity of industrial accidents has led to urban regulation of risk activities in industrial areas. This issue was raised at the earliest stages of industrialization and has been addressed, particularly since the 1970s, by European regulations, i.e., the Seveso Directives and their national adaptation [7]. It is currently a priority in the scientific and technological development in the Russian Federation until 2025 [8]. The European Union (EU) has developed several land use planning (LUP) regulations around Seveso facilities. These regulations follow multiple approaches: deterministic with an implicit judgment of risks; consequence based, risk based, or probabilistic; and semiquantitative [9]. Previous studies have developed new approaches and criteria to assist the LUP implementation in France [10], Italy [11,12,13], Spain [14], Romania [15], and Greece [16]. In Mexico, according to the General Law of Human Settlements, Territorial Ordering, and Urban Development, the location of industrial facilities and the identification and establishment of measures to cushion negative externalities are the responsibility of municipalities. However, when carrying out this task, the municipalities should promote social and citizen participation [17]. Thus, each entity carries out LUP through state laws and regulations. Mexico City does this through the Urban Development Law of the Federal District [18], Guadalajara through the Urban Code for the Jalisco State [19], while Monterrey bases this on the Law of Human Settlement, Land Management, and Urban Development for the State of Nuevo León and its regulations [20]. It is necessary to strengthen the instruments for the implementation of disaster risk reduction and risk assessment. Doing so ensures more sustainable urban planning to accommodate the rapid development that goes hand in hand with the future growth of urban populations [21].

Accidents involving chemicals include explosions and fires. Toxic substances have severe consequences for human health and the environment [22], so their release is a critical public health issue. Chemical accidents that have established a precedent for effective facility regulations include the 1984 release of methyl isocyanate (MIC) gas in Bhopal, India, and the LPG explosion in San Juan Ixhuatepec, Mexico. One connection between these two events, which magnified the number of casualties, was the population density around these facilities [23,24].

The characteristics of the exposed urban systems can magnify or reduce the consequences, based on the level of organization, the emergency response agencies, and the level of social perception and preparedness [25]. Vulnerability is defined as the state of susceptibility to harm from exposure to environmental and social stressors and from a lack of a capacity to adapt [26]. In this context, social vulnerability is an essential factor related to the attributes and situation of a person or group that influence their capacity to anticipate, cope with, resist, and recover [10] from the impact of a hazard.

In [23], the authors identify the degeneration of equipment, a lack of technical training and safety precautions, and the absence of legislation enforcement for site emergency preparedness plans as important factors that increase vulnerability in industrializing countries. Vulnerability analysis is crucial in urban risk assessment, especially in developing countries, where a predominant model is a fragmented city, leading to complex and heterogeneous urban landscapes where socioeconomic groups blend due to reduced space [27].

Indicators used in previous studies to assess human vulnerability include population density [15,28], gender, age, disability, education [24], sensitive population [29], socioeconomic status [30], social fragmentation, dependency structure [31], and coping capacity [32,33]. Human vulnerability assessments are essential to ensure that the equitable rule of governments carries out environmental justice.

Environmental justice (EJ) refers to the obligation of the government to ensure that socially vulnerable segments of the population are not disproportionately affected by adverse environmental impacts (EI) or environmental hazards (EH) [34]. Previous studies have confirmed racial and socioeconomic disparities in pollution and EH distribution [35,36,37]. Principal discrepancies include the location of unwanted land use near minorities and poor people or demographic changes after siting. These discrepancies have led to increased concentrations of minorities and the poor around these sites due to economic, sociopolitical, and racial discrimination [38].

### 1.1. Regulations of Major Hazards and Land Use Planning in Mexico

In Mexico, federal environmental law classifies risk activities into (1) federal high-risk activities and (2) state-level risk activities based on the severity of outcomes for ecosystems and the environment. High-risk activities lead to storage of toxic and flammable chemicals in volumes above those established by the first and second high-risk-activity lists launched in 1990 and 1992.

The urban development legislation requires that municipalities be responsible for promoting and executing actions that prevent and mitigate the risk of human settlements and augment their capacity to recover in the presence of natural and anthropogenic hazards. Urban development plans and programs should also consider resilience when defining the intended use for land and reserves. Development plans are essential documents for presenting strategies that guide future development. Thus, they have the potential to enhance cities’ resilience [39].

One of the main problems in Mexico related to this issue is the lack of connection between industrial regulation and land use planning. Although the guide for environmental risk assessments mentions that identifying risk areas contributes to land use principles at the municipal level, these areas are not established and query mechanisms do not exist. Therefore, the guidelines for the development of land use plans do not mention these risk areas as basic information in municipal regulation. Consequently, relevant information about risk facilities, such as storage volumes of hazardous chemicals and offsite consequence analysis, is often not available at the local level and therefore is not considered during land use planning and facilities land use authorization. A transition to risk-based planning has several challenges, including: how to satisfactorily define acceptable, tolerable, and intolerable risks; how to incorporate the views of stakeholders and affected communities; and how to ensure that potentially controversial decisions over land use options are robust and defensible [40].

### 1.2. Industrialization and Mixland Use in Mexican Metropolises during the 20th Century

In the three largest Mexico cities, industrialization and mixland use are more relevant due to the urban growth and fragmentation that occurred during the 20th century and the first part of the 21st century. The reason is the complex interactions between industrial activities, workforce demand, residential real estate market pressures, and a tendency toward conurbation along transport corridors that made up the Latin-American pattern of urban development [41,42]. Low-income and informal residential zones developed due to real estate housing deficits and a rural–urban crisis [43], leading to social housing developments, satellite suburbs, and slums.

Macroeconomic national and international interactions at the regional level led to nearby cities [44], industrial development into transport corridors and urban peripheries [45], or inter-industry specialization and policy-driven clusterization rather than favoring competitive/comparative advantages [46]. Urban forms have significant implications for social, ecological, and economic city functionalities and can play a crucial role in enhancing their resilience and sustainability [47].

Urbanization refers to the population flow from rural to urban areas that began during the Industrial Revolution, when workers moved from agricultural areas to cities to obtain jobs in factories and/or gain access to urban services [48]. This process involved the appropriation of agricultural land to produce non-agricultural goods, which is a core part of urban sprawls and represents a constant expanse of urban land and the formation of satellite spaces with several complex land use dynamics.

The development of the industry sector and the modes of production were the main factors that determined the territory organization. The location of industries was key, as industries sought to minimize costs and maximize profits through the availability and proximity to energy sources and raw materials. Therefore, manufacturing was subject to development in rural areas, not urban centers, which is not always the case in current urban dynamics.

Industries embedded in a global system of production depend, to a lesser extent, on these factors, and they produce new urban forms because they rely on multinational software and electronic corporations, government entrepreneurial activities, and amenities valued by the information-age elite [49].

In his book, Villareal describes this process for Mexico. Industrial capitalism consolidated in 1876 with the prominence of the textile industry. With steam, and later electrical energy, territorial dependence on water currents ended, making it possible to separate manufacturing companies from natural resources. Road networks developed in all directions, and the first Mexican rail lines reduced transport times and costs. At the beginning of the 20th century, a new pattern of territorial distribution emerged. Mexico City expanded due to an economic policy that eliminated taxes on goods produced by other entities and attracted foreign investments in mining, railway, and real estate located in privileged spaces, thus strengthening the centralization of the city. The national armed struggle between 1910 and 1930 led to an economic crisis due to a weakening of productive forces, destruction of infrastructure projects and resources, loss of foreign investment, workforce demolition, and private production [50].

Import substitution industrialization (ISI) policies guided the economy during this period, transforming Mexico from a mainly agricultural economy with a rural population to a semi-industrialized and highly urban country with steady economic growth. The development was based on decisive government intervention and a clear public policy that promoted manufacturing for export and supply chain diversification during the past three decades [51]. Small urban spaces were mixed with industrial corridors, providing a nearby workforce and consequently contributing to economic development, which was imperative to sustaining the national economic policies.

The years from 1980 to 2010 were challenging for the national economy in terms of per capita income, gross domestic product, and annual manufacturing growth. During these decades, the per capita income staggered at USD 1000/year, while the gross domestic product and yearly manufacturing growth barely went over 5% [52]. During this time, the North American Free Trade Agreement (NAFTA) increased international trade rapidly, while decreasing export taxes. Government regulation of prices for intermediate materials reduced hand in hand with temporary import policies that benefited global manufacturing in several areas: primarily automobiles, textiles, shoes, electronics, steel, canned products, and petrochemicals. This led to a further increase in industrial activity and the dispersion of industrial zones to connect supply chains to road or rail corridors, leading to a dependence on international markets [51]. Furthermore, the growth of major cities that integrated small urban centers into their structure without a formal urban planning strategy led to a mixture of environmental risk areas (ERA)—industrial activities and residential zones—that directly reflected the regional dynamics [42].

These complexities clarify the interactions between population, risk, and marginalization as some of the critical elements that form a reference framework of urban risk management.

This study evaluates the population and marginalization characteristics of chemical risk scenarios to understand their geographic relationships and assesses environmental justice approaches to the spatial distribution of these phenomena.

### 1.3. Area of Study

Mexican metropolitan areas are defined as a group of municipalities that interact around a major city with more than 50,000 inhabitants. The metropolitan area of Mexico City comprises Mexico City and 60 adjoining municipalities, with an area of 5954 km^2^. Mexico City is one of the most populated cities in the world; in 2020, the population was around 22 million. The main economic activities are commerce, financial and insurance services, transport, and tourism.

The Monterrey metropolitan area, in northeast Mexico, is formed by Monterrey City and 17 municipalities. It is the second-most populated area in Mexico, with more than 5.3 million inhabitants in an area of 7657 km^2^. The main economic activities in the Monterrey metropolitan area are services and manufacturing.

The Guadalajara metropolitan area is located in central Mexico and comprises Guadalajara City and 10 municipalities with 5.2 million inhabitants in an area of 2735 km^2^. Its main economic activities are industry and services.

## 2. Materials and Methods

### 2.1. Chemical Hazard Assessment Map

Ammonia, chlorine, and liquefied petroleum gas (LPG) were the chemicals selected to develop offsite consequence scenarios in the studied area. The selection considered the storage of high volumes in refrigerated facilities, wastewater treatment plants, and fuel distribution plants, in addition to spatial patterns. Economic activities spread throughout the three metropolitan areas use ammonia storage and, therefore, may be linked to a more extensive urban exposure. In contrast, chlorine and LPG storage located outside urban areas coincide with patterns of marginalization and informal settlements.

The three selected chemicals have several effects on human health. Gas and liquid ammonia are irritating to the eyes (conjunctivitis, calcific band keratopathy, and/or permanent damage), respiratory tract (irritation, laryngospasm, tracheitis, bronchitis, asthma, chemical pneumonitis, or pulmonary edema), and skin (irritation and rashes) [53,54,55]. Low doses of chlorine can cause mild injury to the airways, while high doses can cause dyspnea, airway obstruction, cough, cyanosis, nausea, and vomiting; the eyes, skin, and heart can also be affected [56,57]. Inhaling propane gas (the main component of LPG) causes dizziness, nausea, vomiting, confusion, hallucinations, and feelings of euphoria and suppresses the function of the central nervous system (CNS). Prolonged exposure can lead to CNS damage, nosebleeds, rhinitis, halitosis, oral and nasal ulcerations, conjunctivitis, bloodshot eyes, anorexia, thirst, lethargy, weight loss, and fatigue [58].

Federal risk facilities used the National Statistics Directory of Economic Activities (DENUE), based on the 2018 National Institute of Geography and Information (INEGI) economic census. DENUE provides information about the type of economic activities in Mexico, their geolocation, number of personnel, and addresses [59]. According to the three offsite consequence scenarios analyzed, facilities selection used the North American Industry Classification System (NAICS) (Table 1).

Geolocation of wastewater treatment plants and fuel distribution plants was confirmed using Google Earth.

An alternative-release scenario was established according to the Health and Safety Executive’s (HSE) failure rate and event data for general-pressure vessels, as shown in Table 2.

The American Industrial Hygiene Association (AIHA) Emergency Response Planning Guidelines evaluate the distance to the endpoint of alternative scenarios. Table 3 shows the results of modeled scenarios.

Emergency Response Planning Guidelines (ERPG) show the estimated concentrations nearly all individuals could be exposed to for up to 1 h without mild or transient adverse health effects (ERPG-1), irreversible or other serious health effects or symptoms (ERPG-2), or life-threatening health effects (ERPG-3) [60]. In thermal radiation consequence scenarios, LOC-1 is related to pain within 60 s, LOC-2 is associated with second-degree burns within 60 s, and LOC-3 is potentially lethal within 60 s [61].

The Environmental Protection Agency’s (EPA) ALOHA software was used to determine the distance to the endpoint of alternative scenarios. ALOHA uses a Gaussian dispersion model to describe neutral buoyant gas dispersion using meteorological conditions of Table 4. The areas potentially affected in the modeled scenarios were calculated using GIS software ArcMap 10.2.2 (ESRI, Ottawa, Ontario, Canada).

### 2.2. Vulnerability Assessment

The Urban Marginalization Index (UMI) developed by the Mexican Population Council (CONAPO) [62] was used to identify population sectors that exhibit deficiencies due to social inequalities. This index groups different indicators into four dimensions using principal component analysis, as shown in Table 5.

The analysis employed the UMI at the local disaggregation level using basic geostatistical areas (AGEB). An urban AGEB is a geographic area occupied by a set of blocks perfectly delimited by streets, avenues, walkways, or any other easily identifiable feature of ground and whose land use is mainly residential, industrial, services, or commercial.

### 2.3. Analysis

Metropolitan areas from the national urban system were taken as an initial spatial unit and then adjusted to conurbation based on data availability, continuity of spatial sampling units, and road connectivity.

**Figure 1:**
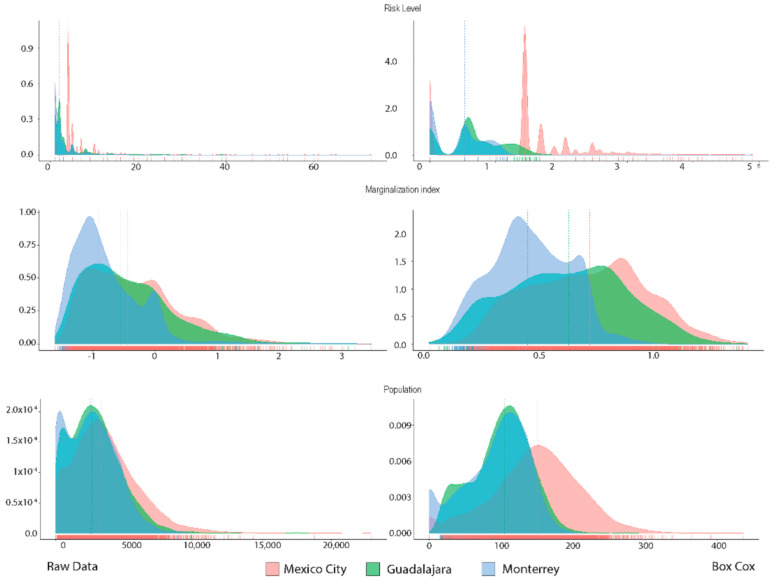
Raw and transformed data for risk level, marginalization index, and population.

Descriptive statistics were calculated (Table 6), observing left skewness for all variables in each city in a similar pattern, so Box–Cox data transformation was used to adjust data into a normal distribution (Figure 1) to optimize the geostatistical models according to specific lambda values for each dataset.

The experimental semivariogram and variogram were calculated for single variables and pairs of variables, respectively, using the transformed data (Table 6) in the ArcGis 10.2.2 geostatistical extension. Each experiment compared univariate and bivariate theoretical and empirical models, calculating nugget, partial still, and range values adjusting LAG distances until an optimal model was obtained.

## 3. Results

### 3.1. Guadalajara City

**Figure 2:**
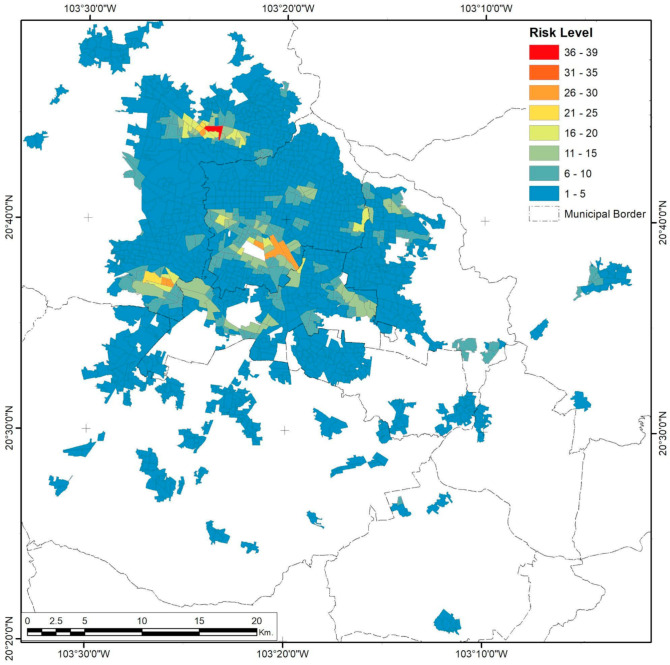
Guadalajara City risk level by AGEB. Developed by the authors.

**Figure 3:**
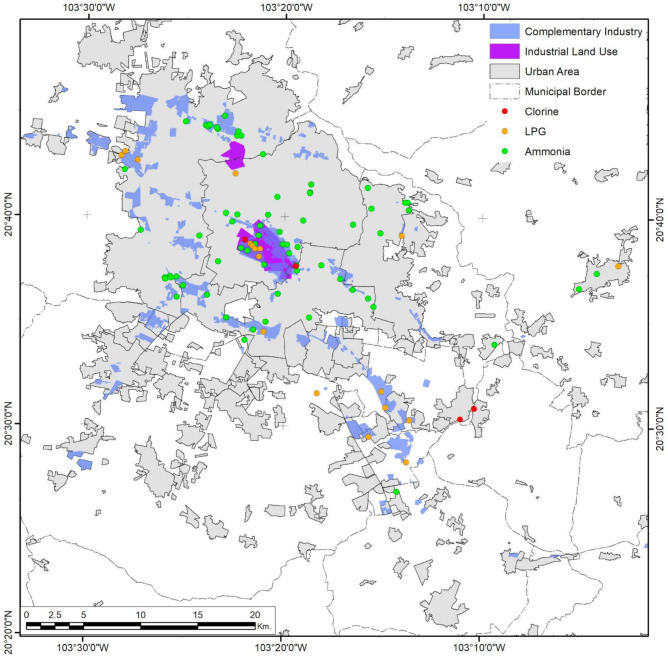
Guadalajara City urban area. The presence of industrial risks and their spatial relationship with industrial coverage. Developed by the authors with data from [59,63,64,65].

Guadalajara City has a specific center–periphery urban growth, reflected in the three variables studied. Risk levels form small, continuous east–west corridors in relatively compact areas (Figure 2), due to not only the dispersion of pollutants but also clusters of industrial activity and spatial population distribution. The location of selected federal risk facilities in Guadalajara shows that only 46.7% are in industrial areas (Figure 3).

**Figure 4:**
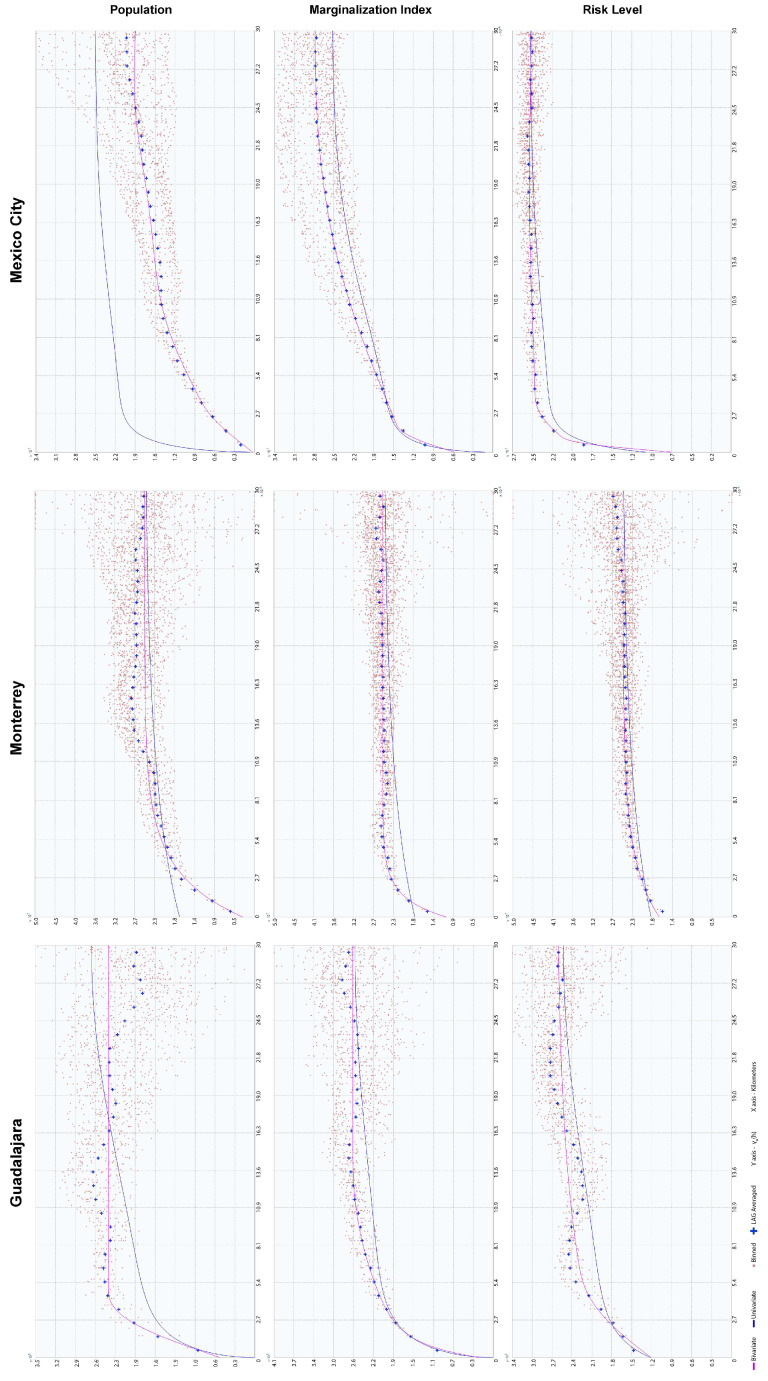
Semivariograms of each city and variable.

According to the estimated maximum range of the semivariogram (Figure 4) for this variable, the reach of the continuously decreasing risk tends to spatially correlate in 5.2 km ranges. The exception to this pattern is a risk corridor related to the south bypass road that extends to the west city limit, in the southern border of the Zapopan and Tlajomulco de Zúñiga municipalities.

**Figure 5:**
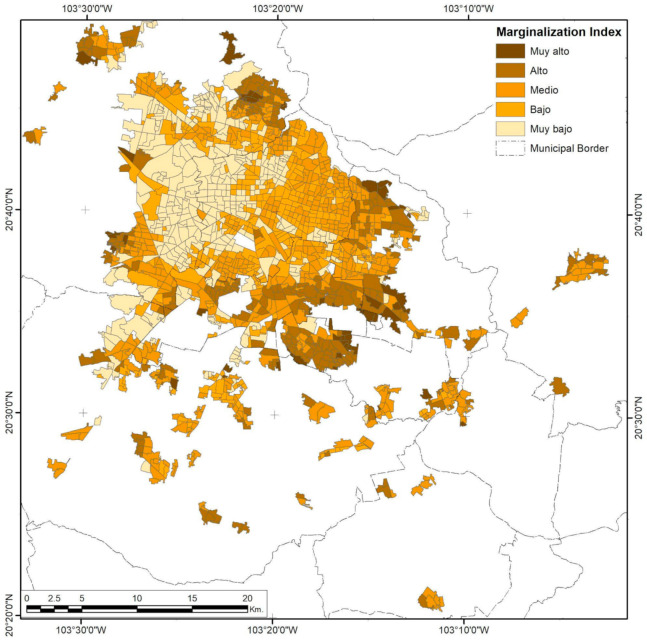
Guadalajara City Urban Marginalization Index (UMI) by AGEB. Developed by the authors with data from [62].

This corridor also divides the very low UMI into a centric pattern with high spatial clustering and a south-west satellite cluster (Figure 5), formed mainly through high-income suburbs and country club residential homes limited in the east by Federal Highway 80, with a mid-UMI cluster within the city limits. There is also a significant concentration of a very low UMI in the centric west part, while low and mid UMIs form a ring, mixed tightly together, that follows the main roads to the city highway exits. The high and very high UMIs are concentrated in peripheral AGEB units, mainly in the southeast part of the metropolitan area, further away from the bypass road.

The UMI semivariogram (Figure 4) shows that this autocorrelation pattern has statistical significance until 7.4 km in the first quintile. Compared to a penta-spherical model, the second and third quintiles have a more comprehensive range of 14.15 and 22.17 km, respectively, i.e., while AGEBs with a low UMI tend to be close to each other, the high-marginalization areas spread out over the city in tight, small clusters but in a constant gradient of exclusion from low-marginalization areas. There is a typical spatial distribution of marginalized spaces, such as slums and informal settlements, in growing cities, with gentrification processes that lead to expulsions [43,66].

**Figure 6:**
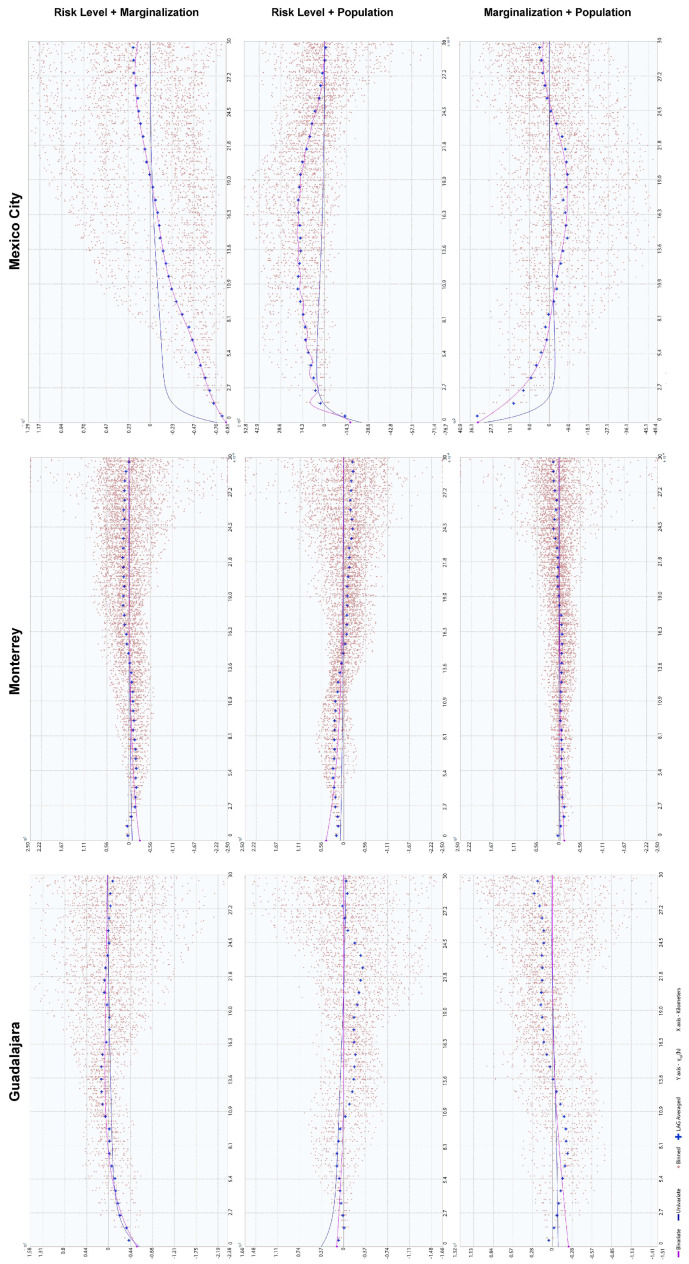
Correlograms of each city and pair of variables.

The risk level–UMI spatial interaction is limited to an extension of 11.19 km, with an average negative correlation limited to a range of 8 km and a wholly negative correlation with only 2.5 km (Figure 6). Therefore, even when the risk is sparse in the urban environment, there is a progressive pattern of correlation, from middle negative to low positive, between the UMI and the presence of risk influenced by regulations. However, marginalization is still related to the risk area borders regardless of the population density in the surroundings.

**Figure 7:**
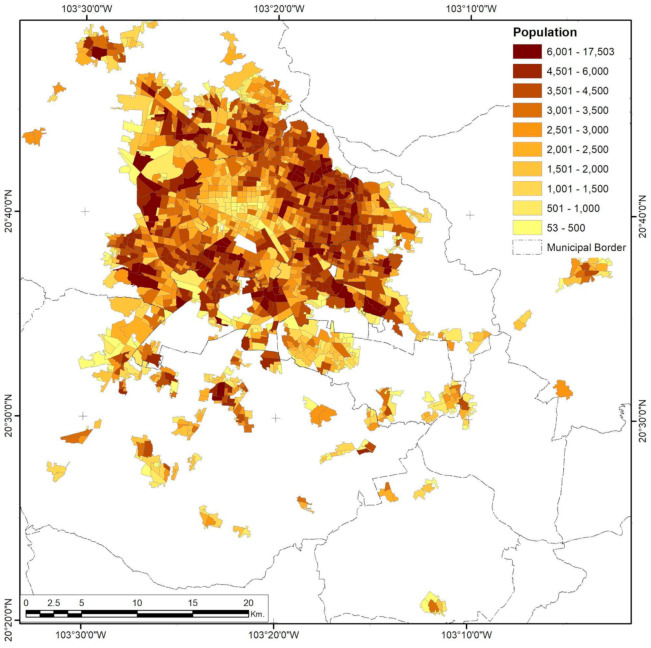
Guadalajara City population density by AGEB. Developed by the authors with data from [63].

The tendency for a close-gated community type of urbanization dating from the 1970s is one key element of the population distribution of Guadalajara (Figure 7). Its interaction with the garden city urbanization design of the mid-1940s to 1970s also adds to the complexity of the variables’ interaction [67,68]. Small-scale densification is isolated from the surroundings, which eventually form larger clusters rather than keeping low-density urban islands. This pattern is seen in the respective semivariogram (Figure 4) through an increased and constant variance until a maximum range of 8.57 km is reached, where spatial autocorrelation is no longer present.

### 3.2. Monterrey City

**Figure 8:**
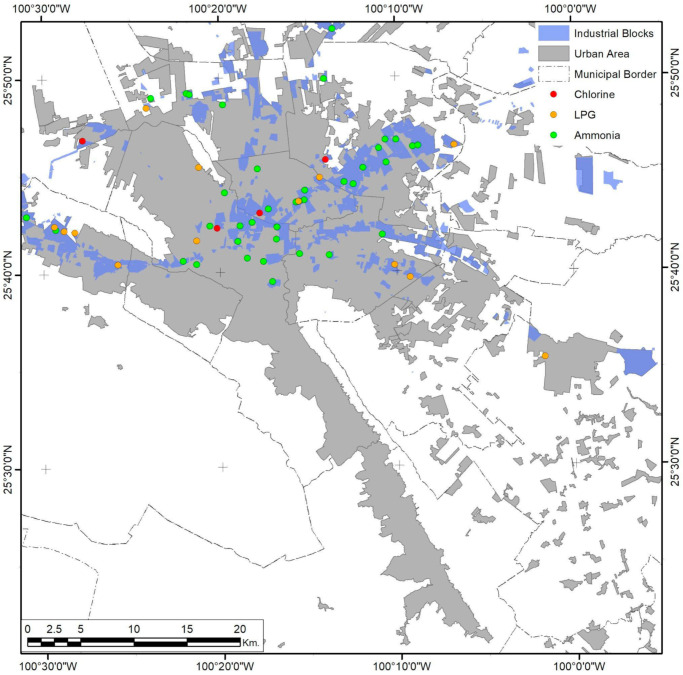
Monterrey City urban area. The presence of industrial risks and their spatial relationship with industrial coverage. Developed by the authors with data from [59,63,65].

**Figure 9:**
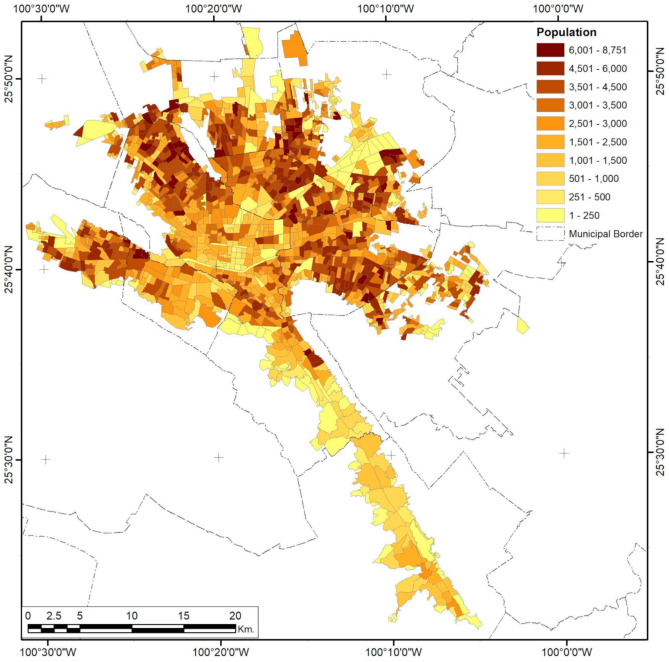
Monterrey City population density by AGEB. Developed by the authors with data from [63].

Analysis of federal risk activities in Monterrey City shows that only 51.72% are in industrial zones of the metropolitan areas (Figure 8). The three variables studied show a completely different spatial distribution compared to Guadalajara. While both cities have had sprawling processes, the way the population of Monterrey scattered into the urban form created urban sectors limited by topological constraints. Meanwhile, highway connectivity was concentrated in the northern parts of the city, leaving the downtown area unpopulated to a great extent (Figure 9). The southernmost corridor and some parts of the central area of the metropolitan urban areas also have low population densities.

Population corridors have a maximum range of 11.70 km and tend to be dense in the inner parts of theory corridors but have a low population density in their extremes, either in the urban borders or in regions that connect with the central urban space (except for the southeast corridor, where a sustained low density is present). Density differences are balanced, with minimum variation in the first 3.0 km and minor variation increments from this distance to the maximum range, where variations start to become incrementally sparse, as shown in the semivariogram for this variable (Figure 4).

**Figure 10:**
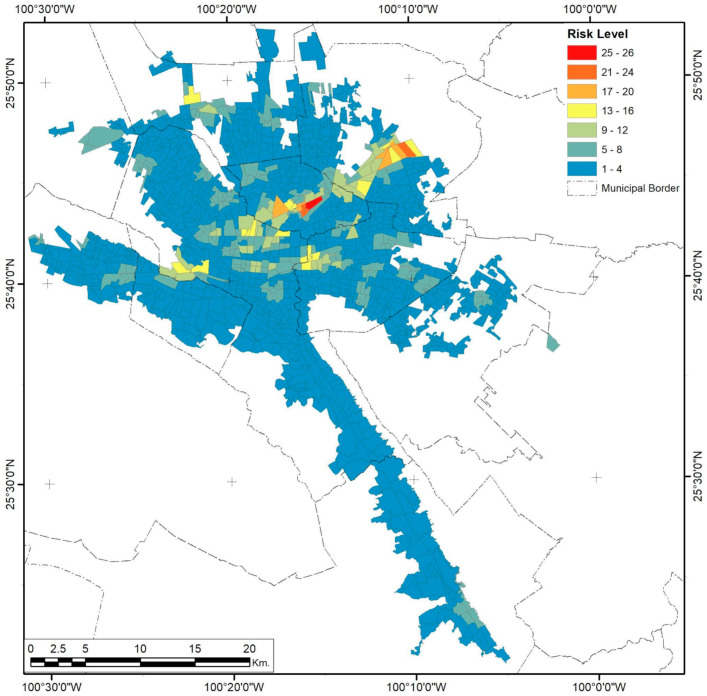
Monterrey City risk level by AGEB.

Urban clusterization of risk levels follows a clear transversal path to the north and west urban corridors, connecting the extreme southeast of the city with its western corridor through the northern part of the city center (Figure 10). A secondary corridor with lower risk levels forms, connecting the downtown regions of the Santa Catarina, Monterrey, and Guadalupe municipalities (Figure 10), and borders the southern part of the urban city space. The spatial consistency of this phenomenon is present in a maximum range of 15.03 km, with low variations in the first 2.1 km, which means that some risk levels tend to be together and mutually isolated between extreme higher values. Still, mid-risk values are cohesive, thus backing up the corridor’s spatial distribution pattern.

**Figure 11:**
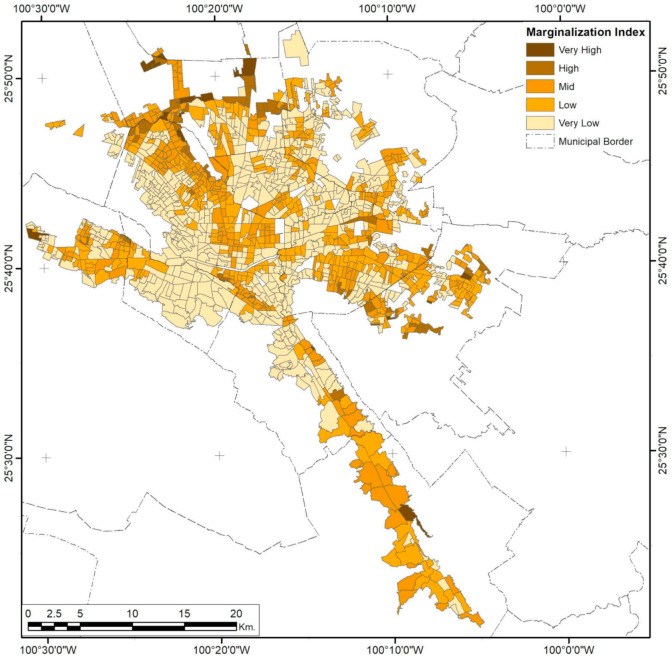
Monterrey City Urban Marginalization Index (UMI) by AGEB. Developed by the authors with data from [62].

Marginalization patterns are also affected by corridor sectorization (Figure 11), but spatial variation increases rapidly as the distance increases, making spatial consistency meaningless for distances higher than 4.04 km. LAG differences show an exponential pattern of variation inside this range, and the maximum UMI is exceptionally high in the urban borders of isolated AGEBs that do not share boundaries.

The risk level–UMI correlation is minimal. Only in the 4 to 6 km range does there exist a little negative mean correlation between both variables (−0.019), but it is not enough to sustain interaction between both phenomena. A similar relationship is found in the risk level–population correlation (Figure 6). Between 4 and 8 km, eigenvector values show a minimal positive correlation (0.031). This also applies to UMI–population correlations, with a minimal negative correlation in 2 to 3 km eigenvector values (−0.014).

This means that the three elements studied are not deeply related or that Monterrey City has a more complex process of urbanization and social segregation [69] than can be observed in detail by UMI components, but further analysis could better address some low spatial correlations.

### 3.3. Mexico City

**Figure 12:**
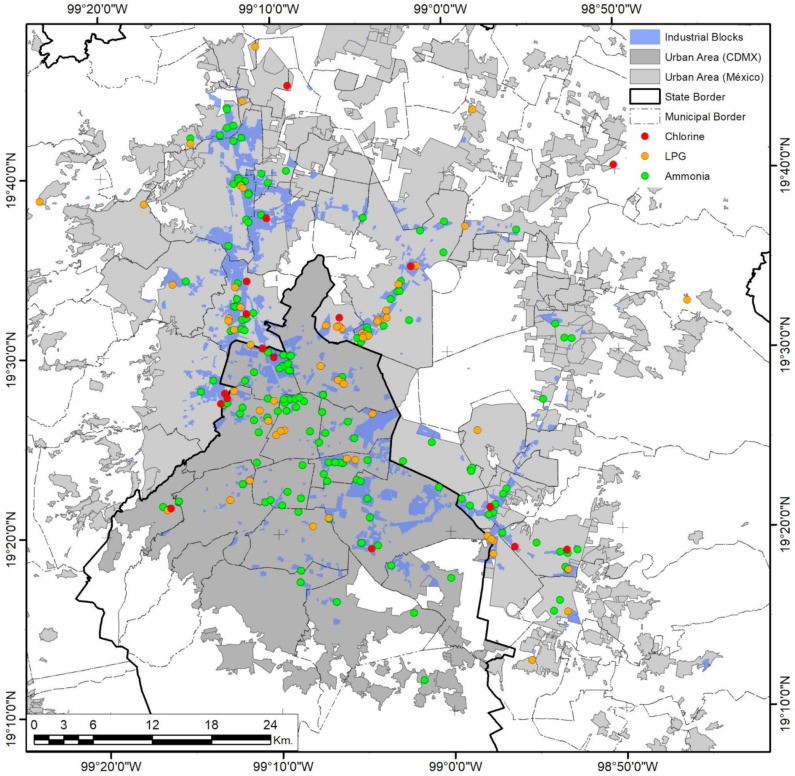
Mexico City urban area. The presence of industrial risks and their spatial relationship with industrial coverage. Developed by the authors with data from [59,63,65].

**Figure 13:**
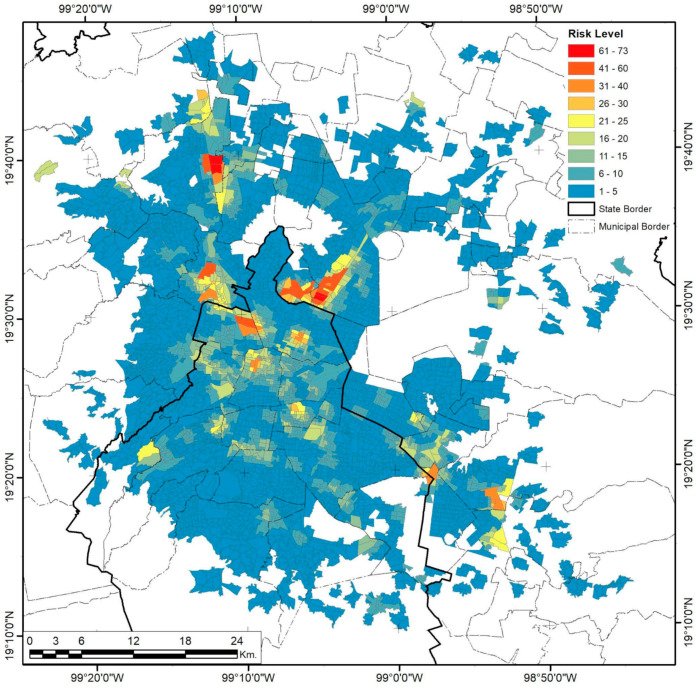
Mexico City risk level by AGEB.

As the biggest and most complex city in the country, Mexico City has undergone several sprawling and densifying processes, evident in its urban form and population distributions. Analysis of high-risk facilities shows that only 58.8% are inside industrial areas (Figure 12). De-industrialization has been part of the public territories policy since the 1970s in a constant expulsion of industrial activities, migrating them from the Federal District to the nearby municipalities of Mexico and exerting enormous productive pressure on immediate rural and urban areas [70]. Nevertheless, the urban sprawl and regional metropolis eventually re-integrated the new industrial clusters into the urban fabric in more complex ways, mainly because those industrialization spaces were now dependent on suburban peripheral developments and urban subcenters. Furthermore, industrial activities did not completely relocate during this process; some remained even after the 1993–2008 NAFTA intern ational integration [45]. This explains the cluster and corridor patterns in risk levels (Figure 13), with higher levels inside industrial zones, while mid-level risks tend to follow the road infrastructure that connects them to the more residential areas. The spatial correlation of this continuity is limited to 4.34 km for low variance and 14.64 km for mid-variance according to the respective semivariograms in Figure 4.

The risk level–UMI spatial covariance is significantly similar in different distance ranges up to a 9.61 km limit, with a penta-spherical model, according to the respective correlograms (Figure 6). However, more significant autocorrelations can be obtained for the joined variance fit in a penta-spherical–Gaussian–spherical model, with statistical significance for a 28.05 km extent. This model has a better fit for the population–risk level correlogram, showing a closer relationship between the empirical and theoretical correlograms compared. A hole effect is present in a 2.02 km range, with a high negative covariance for the first kilometer but a positive covariance from the second kilometer on and up to a 25.74 km distance range, showing the high population dependence on these services even when ER or EH is present in the vicinity.

**Figure 14:**
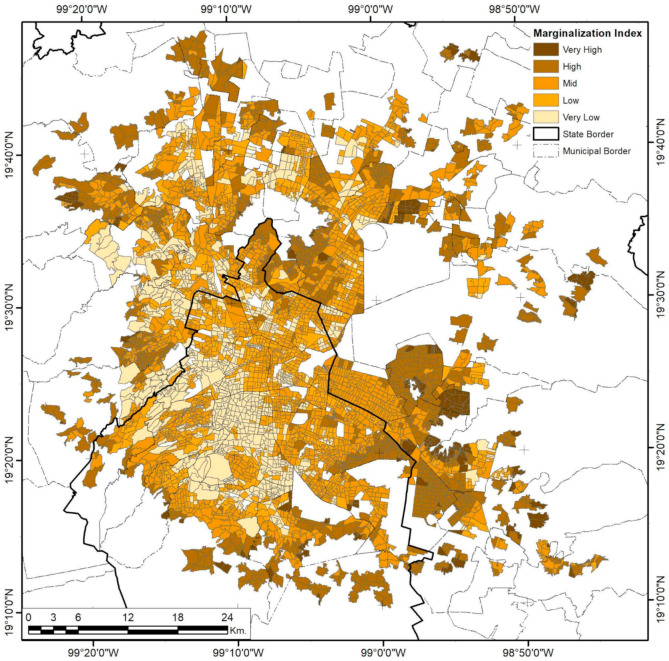
Mexico City Urban Marginalization Index (UMI) by AGEB. Developed by the authors with data from [62].

Like Monterrey City, Mexico City shows UMI trends that form gradients with higher marginalization in the urban periphery (Figure 14). In addition, a low spatial variance in short ranges is observed in the 2.02 km range for the same UMI value, while middle and low UMIs tend to remain constant in corridors and widespread clusters. Long-distance autocorrelation exists in an 18.07 km range, but positive correlations are only present in 8.2 km limits and at distances of 25 km or more, indicating that the same marginalization levels tend to be closer, with fewer variances between bordering AGEBs.

**Figure 15:**
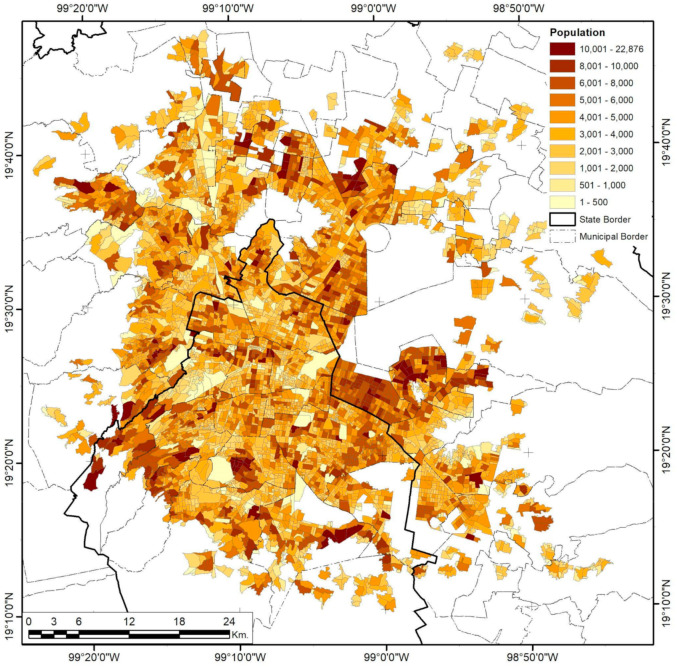
Mexico City population density by AGEB. Developed by the authors with data from [63].

Population density has the most fractured pattern of all the variables studied (Figure 15), with only 1.15~4.14 km ranges of cluster consolidation, while the city as a whole presents a 26.49-km-wide hole effect, mostly because of the presence of a central business district (CBD) and its importance for the national and local economy.

## 4. Discussion

The industries selected to evaluate the offsite consequence analysis in this work are relevant, considering that LPG and wastewater treatment plants are typically in the outskirts of urban areas. Meanwhile, ammonia facilities are widespread, not limited to urban peripheries or industrial zones.

The three cities studied have entirely different growth patterns, risk locations, urban marginalization, and populations. Nevertheless, there are consistent patterns of spatial exclusion for marginalized areas in the urban periphery that do not always correlate with risk levels or the population in short distances. Still, they reflect the complex development of the industrial sprawl and activity [23].

Mexican water treatment is a heterogeneous issue that comes within the limits of both state and urban administration. There is national legislation regarding it and a decentralized institution to regulate national water management, but the operational issues remain under local jurisdiction. Since 1994, the water treatment capacity has tripled in volume. In 2011, about 97.6 cubic meters per second were treated at the national level, and the overall capacity limit was 137 cubic meters per second [71]. However, the national goals did not meet the milestones set, primarily because of infrastructure delays and operational limitations. The goals for 2015 estimated that only 69.4% of the total effluent volumes were treated, and a new set of facilities was needed to be embedded within urban areas in order to increase these volumes.

Nuevo León, the state in which the Monterrey metropolitan area is, was one of two states with 100% wastewater treatment coverage up to 2011, despite having only four large facilities inside the metropolitan area and a few in the peripheral unurbanized areas. These latter facilities were not included in this study because their risk dispersion did not affect the AGEBs. However, the reality in Jalisco, Mexico State, and Mexico City is different, with only 36.8%, 27.4%, and 15.1% wastewater treatment coverage, respectively. These values indicate that both cities should address wastewater treatment facilities in their urban areas and, more importantly, in the major metropolitan areas. Different scenarios should be considered to include metropolitan growth and informal settlement integration/regulation for an integral urban planning approach.

Ammonia risks are present in industrial zones and also in more centric locations in cities such as Guadalajara and Mexico City. These facilities can be considered as those connecting the risk corridors, even when they are not attached to industrial land use zoning; however, there is definitely an economic interaction explaining their strategic location.

Population disaggregation and its interaction with risk distribution also raise concerns that should be addressed by urban planning policies. The three cities analyzed have positive covariances in the estimated hazard dispersion areas that exceed risk dispersion distances, with Mexico City being the most problematic. High covariance values continually increase over long distances from the hazard-affected areas, forming corridors with complex interactions that oscillate in a 9.6 km range but maintain a Gaussian covariance pattern up to a 28.06 km range. This pattern is a clear example of how risk-related areas expand, interconnect, and surpass industrial zones and designated land use areas [5]. Furthermore, this pattern adds complexity to the constant exposure to environmental hazards in transport corridors that are already present in the city [72] and tightly bound by commuting and industrial zones.

Mexico City also faces significant challenges related to chemical risk management, due to urban gentrification and the pressures of peripheral, highly competitive economic poles, thus reducing safety buffers within high-exposure ranges, even when there is no correlation with high marginalization [70].

In the analyzed cities, only 5.1% of social-environmental conflicts are related to very high urban marginalization, 34.6% are related to mid-marginalization, and 26.9% to low marginalization. Most of these conflicts are related to water scarcity, urban waste disposal, and irregular disposal of high-toxicity substances, in that order. Overall, even when identified environmental conflicts are present in the urban space, potential environmental hazards are not considered as part of urban territory planning or integral urban management policies. This is either because risk management is not properly addressed or because urban pressures are more intense for facilities needed for urban functionality and market accessibility, especially in the periphery of global cities and even more so in the global south [73].

Guadalajara and Monterrey do not present these kinds of pressures despite being the second and third main cities in Mexico. The significance for covariance ranges is wider, even reaching the urban borders of both cities. This is not the case for Mexico City, where negative values are in the first kilometer. A low positive correlation is present in Guadalajara and Monterrey, and a negative correlation appears only after 10 and 13 km, respectively, meaning that facilities are considered centric spaces on a broader range and the population tends to densify in distances between 5 and 8–11 km from the risk locations. The real estate and housing markets are present in these areas despite the presence of chemical risk sources that make the neighborhood less desirable and decrease the overall value of its properties [36]. These elements can be explained by economic attractiveness and metropolization [74] or sprawling that keeps industrial activities clustered even after de-industrialization public policies are implemented, mostly because of technological specialization [75,76,77]. If these problems are left unchecked or not considered in land use planning, Guadalajara and Monterrey will face these problems in the near future, with the same or worst consequences than in Mexico City.

Guadalajara has also seen industrial migration to surrounding municipalities, mainly due to the increasing value of housing and close-gated community development [68]. While this explains the lack of interaction between industrial risk areas and the population variables studied, it does not prevent further interactions between variables, especially those that cause safety areas to be urbanized. In addition, even when there are several high-marginalization clusters in the city, they are mutually exclusive and isolated in the periphery, where new risk-related facilities can be established in the future.

Forced commuting dynamics, automobile dependency, and public transport connectivity also should be considered [78] in order to better understand the complex interaction of industrial risks, marginalization, and the location of commercial services in the metropolis, because future scenarios based on the present analysis could lead to suburb dynamics where industrial and environmental risks can emerge without proper land use regulations, deliberate EH concentration for specialized competitiveness, or even more complex issues between ER and transport-related environmental pollution.

Trends in population density are explained by the heterogeneous growth model of Mexican cities [79] (extensive and fragmented) and the deployment of multiple specialized centralities [77]. The highest population densities are now in peripheral population centers and clustered, close-gated housing development areas with their own peripheries where new marginalized spaces (such as slums and informal settlements) tend to form. According to [76], one primary attribute of Latin-American capitalist development has been the constant presence of a whole urban economic sector constituted by a great mass of the unemployed, underemployed, self-employed, or precariously employed, known as the informal sector. This should be considered as the main social group affected by unregulated urban dynamics, as well as the one that may eventually suffer the consequences of a territorial planning policy that hides environmental risks.

## 5. Conclusions

This paper evaluated spatial patterns of the potential hazards of ammonia, chlorine, and LPG in three main Mexican metropolitan areas: Mexico City, Guadalajara, and Monterrey. Clustering of such hazards in the study areas is related to the industrial corridors that permit the access and exit of materials and finalized products. Marginalization areas in Mexico City and Monterrey show gathering trends, followed by exclusion tendencies between low and high indexes and the expulsion of these to the limits of the urban sprawl.

Incorporating anthropogenic risk analysis, especially that of a chemical nature, into the urban planning process is significant in guaranteeing the safety of the inhabitants of urban settlements. Even when hazards are not always spatially correlated, specific sectors of the studied metropolitan areas with a medium to low marginalization index and a high-population-density area are potentially exposed to chemical hazards. These must be subject to integral risk management strategies through a close collaboration between federal and local levels to prevent, regulate, control, and respond to emergencies due to environmental risks related to industrial activities.

Sprawling and conurbation play a crucial role in how risks are incorporated into the urban space based on social, economic, and spatial elements. Marginalization and the population do not always explain these patterns, but they definitely interact with environmental and industrial risks in several ways, e.g., the tendency to reduce safety buffers, depending on urban pressures such as gentrification, informal settlements, economic dynamics, urban restructuring, a lack of integrated planning, and topological constraints. Nevertheless, geostatistical analysis can make a substantial approximation for understanding key variables and their spatial interaction. This would establish more specific methodologies involving multidimensional and multiscale approaches to better land use planning and territorial development programs in areas with early sprawling and metropolization, as well as in already consolidated urban areas where the real estate market could try to develop and industrial risks are already present.

Mexican territorial planning tends to create environmental risks outside of the planning process. However, the key elements of land use planning and urban forms should be tackled in an integral manner. For example, issues such as fixed air emissions should be included as latent hazards in the set of human exposure risks considered for territorial planning, commercial activity location, housing, and transportation.

All three of the studied cities should consider safety buffers for the industrial hazards analyzed in order to ensure negative covariances between risk levels, population densities, and marginalized areas, in at least a 5 km range. Ideally, future land use planning instruments should also consider implementing strategies for relocation of industrial clusters related to environmental risks in order to ensure resilience for their cities. This can be possible by creating risk corridors spatially separated from residential areas and new housing developments or by reducing the level of hazard that each industry represents to its surroundings. Implementation of emergency systems should also be considered in areas where economic and urban agencies limit relocation processes to avoid further developing marginalized areas with industrial activities.

How these issues will affect urban growth and land use change depends on how these approaches are included in future territorial planning and urban policies. Effects at the local level (neighborhood and urban corridors) and the necessary changes required to address those effects requires additional analysis, longitudinal approaches, and sub-regionalization based on observed patterns in the semivariograms (Figure 4) and correlograms (Figure 6). These will be the next steps taken in future studies, which may eventually lead to preventive management strategies for industrial environmental risks, their impact on the urban structure, and their impact on the population.

## Figures and Tables

**Table 1:** NAICS classification of facilities studied.

Substance	NAIC Code	Description	Number of Personnel
Ammonia	311511 312111 312113 312120 493120	Production of milk Production of soft drinks Fabrication of ice Production of beer Refrigerated storage	≥250
Chlorine	221311 221312	Treatment of water carried out by the public or the private sector	≥250
LPG	468413	LPG retail trade	≥250

**Table 2:** Hazard scenarios modeled.

Substance	Storage	Release Source	HSE Failure Probability	Thread Modeled	Level of Concern (LOC)
Ammonia	Horizontal tank Volume: 5000 L Length: 3.070 m Diameter: 1.620 m Pressure: 13 kg/cm^2^ State of chemical: liquefied gas	19 mm valve failure	3 × 10^−2^	Toxic gas dispersion	ERPG-1 > 25 ppm ERPG-2 > 150 ppm ERPG-3 > 1500 ppm
Chlorine	Horizontal tank Volume: 730 kg Diameter: 0.762 m Length: 2.02 m Pressure: 5 atm State of chemical: liquefied gas	6 mm hole	4 × 10^−5^	Toxic gas dispersion (heavy)	ERPG-1 > 1 ppm ERPG-2 > 3 ppm ERPG-3 > 20 ppm
LPG	Vertical tank Volume: 110,000 L Diameter: 3.378 m Length: 14.078 m Pressure: 17 kg/cm^2^ State of chemical: liquefied gas	Boiling liquid expanding vapor explosion (BLEVE)	1 × 10 ^−5^	Thermal radiation from fireball	LOC-1 2 kW/m^2^ LOC-2 5 kW/m^2^ LOC-3 10 W/m^2^

**Table 3:** Distance to endpoint of modeled scenarios.

Substance	Level of Concern	Distance to Endpoint		
		Ammonia	Chlorine	LPG
Monterrey	LOC-3 LOC-2 LOC-1	551 m 2.0 km 4.1 km	543 m 1.5 km 2.6 km	482 m 680 m 1.1 km
Guadalajara	LOC-3 LOC-2 LOC-1	531 m 1.9 km 3.9 km	571 m 1.5 km 2.7 km	506 m 714 m 1.1 km
Mexico City	LOC-3 LOC-2 LOC-1	525 m 1.5 km 3.1 km	704 m 1.9 km 3.4 km	495 m 698 m 1.1 km

**Table 4:** Meteorological condition of studied areas.

City	Wind Direction	Wind Speed
Monterrey	East	3 m/s
Guadalajara	East	2.5 m/s
Mexico City	Northeast	1.6 m/s

**Table 5:** Indicators used in the Urban Marginalization Index [62].

Dimension	Indicators	First Component Coefficient
Education	Percentage population from 6 to 14 years that does not attend school	0.101
	Percentage population aged 15 years or over without complete basic education	0.151
Health	Percentage population without access to health services	0.096
	Percentage deceased children of women between 15 and 49 years	0.098
Housing	Percentage inhabited private homes without drainage	0.125
	Percentage inhabited private dwellings without a toilet with a water connection	0.165
	Percentage inhabited private dwellings without piped water	0.151
	Percentage inhabited private dwellings without a floor	0.134
	Percentage inhabited private dwellings with some level of overcrowding	0.145
Goods	Percentage inhabited private dwellings without a refrigerator	0.154

**Table 6:** Descriptive statistics of analyzed variables and their normalization process for Guadalajara (GDL), Monterrey (MTY), and Mexico City (CDMX).

		Pre-Processed Data			Box–Cox Normalization		
		IMU	RiskLv	POP	IMU	RiskLv	POP
GDL	Min.	–1.54	1.00	53.00	0.00	0.00	12.73
	1st Q.	–0.98	1.00	1389.00	0.41	0.00	74.56
	Median	–0.56	2.00	2648.00	0.61	0.58	104.05
	Mean	–0.43	3.382	2813.00	0.60	0.62	99.89
	3rd Q.	–0.02	3.00	3927.00	0.79	0.84	127.40
	Max.	3.47	39.00	17,503.00	1.34	1.67	273.22
	Std. dev.	0.73	3.74	1902.93	0.25	0.46	40.04
	λ (lambda)	N/A	N/A	N/A	–0.3434	–0.5051	–0.3434
MTY	Min.	–1.61	1.00	1.00	0.00	0.00	0.00
	1st Q.	–1.16	1.00	983.00	0.32	0.00	62.30
	Median	–0.91	2.00	2,482.00	0.42	0.53	100.64
	Mean	–0.76	2.60	2,526.00	0.43	0.39	91.68
	3rd Q.	–0.46	3.00	3,785.00	0.56	0.74	125.01
	Max.	3.21	26.00	8,751.00	0.90	1.17	191.93
	Std. dev.	0.58	2.76	1,775.86	0.16	0.39	44.49
	λ (lambda)	N/A	N/A	N/A	–0.8687	–0.7879	0.5051
CDMX	Min.	–1.6	1.00	1	0.00	0.00	0.00
	1st Q.	–0.92	4.00	1901	0.49	1.44	110.80
	Median	–0.44	4.00	3291	0.70	1.44	150.10
	Mean	–0.33	2.40	3605	0.69	1.37	147.40
	3rd Q.	0.08	5.00	4959	0.87	1.69	188.20
	Max.	3.03	73.00	22,876	1.39	4.90	435.80
	Std. dev.	0.73	5.35	2393.63	0.25	0.92	61.63
	λ (lambda)	N/A	N/A	N/A	–0.2626263	0.1	0.5454545

## Data Availability

Data are contained within the article.
